# Copper(II) Complex Containing 4-Fluorophenoxyacetic Acid Hydrazide and 1,10-Phenanthroline: A Prostate Cancer Cell-Selective and Low-Toxic Copper(II) Compound

**DOI:** 10.3390/molecules27207097

**Published:** 2022-10-20

**Authors:** Nayara Júnia de Souza Bontempo, Drielly Aparecida Paixão, Paula Marynella Alves Pereira Lima, Deysse Carla Tolentino Barros, Dayanne Silva Borges, Priscila Capelari Orsolin, Isabella Castro Martins, Pedro Henrique Alves Machado, Ricardo Campos Lino, Tiago Rodrigues de Souza, Luana Munique Sousa Ramos, Samuel Cota Teixeira, Raoni Pais Siqueira, Luiz Ricardo Goulart Filho, Wendell Guerra, Robson José de Oliveira Júnior, Thaise Gonçalves de Araújo

**Affiliations:** 1 Instituto de Biotecnologia, Universidade Federal de Uberlândia, Uberlândia 38400-902, Brazil; 2 Instituto de Química, Universidade Federal de Uberlândia, Uberlandia 38400-089, Brazil; 3 Laboratory of Cytogenetic and Mutagenesis, Centro Universitário de Patos de Minas, Patos de Minas 38700-207, Brazil; 4 Instituto de Ciências Biomédicas, Universidade Federal de Uberlândia, Uberlandia 38405-318, Brazil

**Keywords:** prostate cancer, chemotherapy, copper(II) complexes, cell proliferation, *Drosophila melanogaster*

## Abstract

Prostate Cancer (PCa) is the second leading cause of cancer-related deaths among men worldwide. The treatment of advanced cases is based on chemotherapy, which lacks specificity and efficacy, due to severe side effects and resistance to the traditional drugs. Copper complexes have shown antitumoral efficacy and low toxicity, being considered a promising class of metal-based drugs for the treatment of malignant neoplasms. Thus, the present study aimed to evaluate the cellular effects of a copper(II) complex with 4-fluorophenoxyacetic acid hydrazide and 1,10-phenanthroline (**1**) on PCa cell lines, as well as the mutagenic/recombinogenic and anticarcinogenic potential of **1** in *Drosophila melanogaster.* PNT-2 (non-tumorigenic), LNCaP (hormone-responsive PCa) and PC-3 (androgen-independent PCa) cells were cultured, and cytotoxicity was assessed using the MTT assay. The expression levels of the proliferation markers Ki-67 and Cyclin D1 were analyzed by flow cytometry. Furthermore, the Somatic Mutation and Recombination Test (SMART) and the Epithelial Tumor Test (ETT) were performed. Complex **1** was selective to LNCaP cells, significantly reducing Ki-67 and Cyclin D1 expression levels. Sub-toxic concentrations of complex **1** were defined by the toxicity test in *D. melanogaster*, and no mutagenic/recombinogenic/carcinogenic effects were observed. Anticarcinogenic potential was observed in *D. melanogaster*, suggesting modulating activity of the complex **1** against Doxorubicin, a drug used as control by its carcinogenic properties. Therefore, complex **1** is a possible starting point for the development of new antitumor agents for the treatment of PCa.

## 1. Introduction

Cancer stems from genetic mutations that give cells the unlimited proliferation capacity, loss of response to growth-inhibiting factors, avoidance of apoptosis, immune escape, metabolic modulation, invasive potential, and induction of angiogenesis [1]. The systemic chemotherapy treatment, although able to control the disease, causes debilitating side effects, besides having its effectiveness reduced in the face of resistance mechanisms that compromise the clinical status of patients [2,3,4]. The number of people diagnosed with cancer increases each year, and nearly 10 million died from the disease in 2020. Epidemiological data are frightening, with more than 30 million cases predicted in 2040 [5].

Prostate Cancer (PCa) is the most common malignancy among men and the cases are usually characterized by an indolent course, which is mainly correlated with late diagnosis and disease progression. Despite new therapies, diagnostic imaging methodologies and robust molecular techniques, PCa remains the third most common cause of cancer-related death among men in the United States [6,7], demanding dedicated efforts to discover new effective drugs. Metal complexes have been highlighted due to their promising biological effects (such as cancer, fungal, and microbial infections), in addition to the physical–chemical properties of their transition metals [2]. Metal ions, such as Fe^3+^ and Cu^2+^, for example, are essential to living organisms and, in this context, subject to modifications in the development of less toxic and more selective prototypes [1]. In fact, copper is a metal found in nature that is essential for enzymes acting in the antioxidant defense of the organism, a fact that makes it feasible for use in metal-based drugs. Additionally, there is evidence that copper is capable of inducing DNA cleavage and nucleic base oxidation by producing reactive oxygen species (ROS) [8,9,10]. Both the cytotoxicity of this metal and its ability to inhibit the growth of tumor cells have already been described [11,12,13], though not yet explored, for PCa.

**Figure 1:**
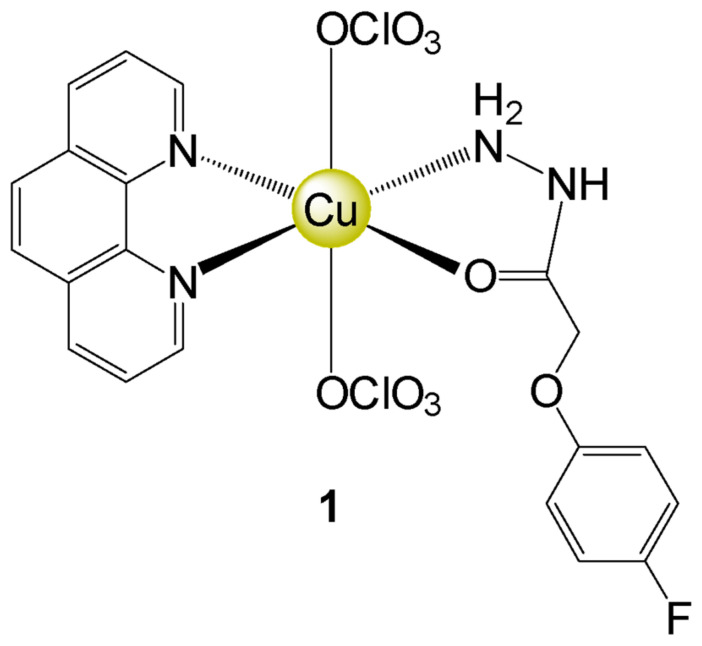
Structure of complex **1** [10].

Paixão et al. (2017) synthesized a copper(II) complex bearing 4-fluorophenoxyacetic acid hydrazide and 1,10 phenanthroline (complex **1**) (Figure 1) [10], which presented DNA binding capacity, as well as antitumoral and antimycobacterial activities. However, it is known that some prototypes may have mutagenic and/or carcinogenic potential, whereas others can mitigate these effects. For this reason, many compounds have been undergoing tests in different experimental systems [14]. In this perspective, tests in *Drosophila melanogaster* are interesting and able to predict the therapeutic potential of different products. Indeed, due to the high similarity to mammalian genes and easy handling and maintenance, *D. melanogaster* is an organism test validated for the study of carcinogenicity, mutation and recombination [15]. The Somatic Mutation and Recombination Test (SMART) is considered as a cheap method capable of generating reliable and reproducible results [16,17], besides being precise in discriminating simultaneously mutagenic, clastogenic, and/or recombinogenic agents. Moreover, it detects the genotoxicity of compounds of different chemical classes and complex mixtures, as well as aerial particles [18,19,20]. The test for the detection of epithelial tumor (ETT) is widely used for the evaluation of carcinogenic or anticarcinogenic activity in an economic, fast, and sensitive way to different treatments. Different protocols are described including experimental designs with isolated or combined compounds, in strategies with pre-treatment, co-treatment and post-treatment assays [21,22,23]. In the present study, the cellular effects of complex **1** were evaluated in PCa cells, and its mutagenic/recombinogenic and antitumoral potentials were assessed in *D. melanogaster*. Regarding the pharmacological and anticancer effects played by essential metals like copper, we hypothesized that complex **1** is a promising prototype in controlling tumor cells.

## 2. Results

### 2.1. Complex **1** Downregulates Proliferation Markers on PCa Cells

**Figure 2:**
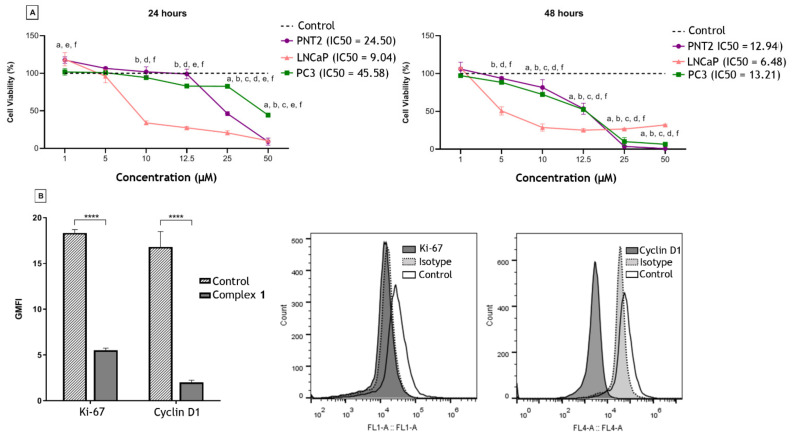
Effects of complex **1** on prostatic cell lines. (**A**) Cytotoxic activity of complex **1** on non-tumorigenic lineage PNT-2 and the tumor lines LNCaP (hormone-responsive) and PC-3 (androgen-independent) after 24 and 48 h of treatment. a: comparison between PNT-2 and control; b: comparison between LNCaP and control; c: comparison between PC-3 and control; d: comparison between PNT2 and LNCaP; e: comparison between PNT2 and PC-3; f: comparison between LNCaP and PC-3. IC50 is also presented. (**B**) Expression analysis of Ki-67 and Cyclin D1 on LNCaP cells by flow cytometry (white peak). Secondary antibody alone was used (traced gray peak). **** *p* < 0.0001. Results were presented as mean ± SD of three independent experiments repeated three times. Control was cells treated with dimethyl sulfoxide (DMSO).

The effects of complex **1** on PCa cells were evaluated by the 3-(4,5-dimethylthiazol-2-yl)-2,5-diphenyltetrazolium bromide (MTT) assay and the mechanism of action recorded by flow cytometry (Figure 2). In LNCaP cells, within 24 h, a dose-dependent effect was observed and, from 25 µM, complex **1** was cytotoxic to PNT-2 (non-tumorigenic). After 48 h the compound was also more active against the LNCaP cells up to a concentration of 12.5 µM. At higher concentrations, complex **1** substantially reduced the viability of the PC-3 cells, although it was also cytotoxic to PNT-2. In Figure 2, the concentration that resulted in 50% inhibition of cell viability (IC50) shows the selectivity of the compound to LNCaP cells with Selectivity Index (SI) = 2.71 and SI = 2.22 after 24 and 48 h of treatment, respectively (Figure 2A). The effect of complex **1** on the proliferation of LNCaP was then analyzed by flow cytometry and the expression of Ki-67 and Cyclin D1 markers was significantly reduced after 48 h of treatment (Figure 2B).

### 2.2. Toxicity Test (TX)

**Figure 3:**
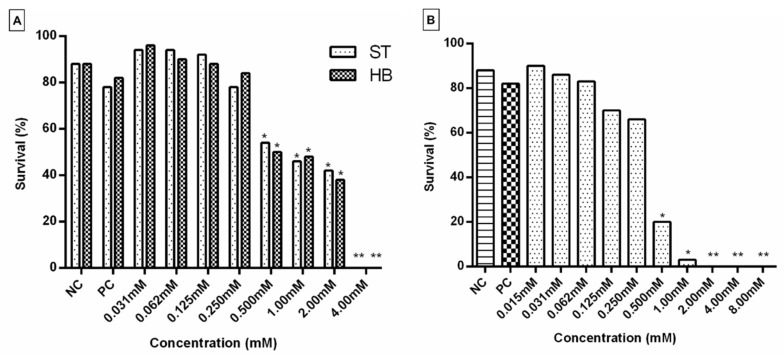
Survival rate of *D. melanogaster* obtained from the Toxicity Test (TX) of different concentrations of complex **1**. (**A**) Lineages of the Standard (ST) Cross and High-Bioactivation (HB) Cross used in the SMART test; (**B**) lineages used in the ETT test. * *p* < 0.05 and ** *p* < 0.01.

Subsequently, complex **1** was evaluated for its mutagenic/recombinogenic and anticarcinogenic potential in *D. melanogaster*. For this purpose, the Toxicity Test (TX) was initially performed with larvae from the SMART and ETT tests in order to define the lethal dose (LD) and the concentrations of the compound to be used later (Figure 3). Concentrations that did not show significant toxicity (0.015, 0.031, 0.062, 0.125 and 0.250 mM) were used in the subsequent assays. When a significant decrease (*p* < 0.05) in the percentage of larval survival was identified, the compound presented a toxicity that interferes with the development of the larvae. Furthermore, the number of emerging adults needs to be enough to conduct the next experiments [24]. In the present study, from the 4.00 mM concentration of complex **1**, no *D. melanogaster* emerged in the SMART assay (Figure 3A), which indicates that this was the DL for the lineage used in the experiment. For individuals used in the ETT test, the DL was 2.00 mM (Figure 3B). These data suggest that the progeny used in ETT was more sensitive to the toxic effect of complex **1** than individuals used in SMART. Otherwise, in both tests, when compared to the negative control, treatments with concentrations above 0.500 mM significantly compromised the survival of the flies. Therefore, these doses were not used in the next experiments.

### 2.3. Somatic Mutation and Recombination Test (SMART)

The SMART test was performed in order to understand whether the treatment with complex **1** may cause some undesirable side effects (mutagenicity/recombinogenicity). Table 1 shows the results of two crosses of individuals treated with complex **1** at concentrations of 0.015, 0.312, 0.062, 0.125 and 0.250 mM, the positive control (Doxorubicin—DXR—0.4 mM) and the negative control (reverse osmosis water). The frequencies of mutant spots of the marked trans-heterozygous individuals from the standard cross and the high-bioactivation cross were also recorded. In none of the concentrations tested, in descendants of the Standard (ST) and High-Bioactivation (HB) crosses, there were significant differences in the total frequency of spots compared to the negative control. Our results indicated the absence of a mutagenic/recombinogenic effect of complex **1** in somatic cells of *D. melanogaster*, suggesting a possible selectivity of complex **1**.

### 2.4. Epithelial Tumor Test (ETT)

**Figure 4:**
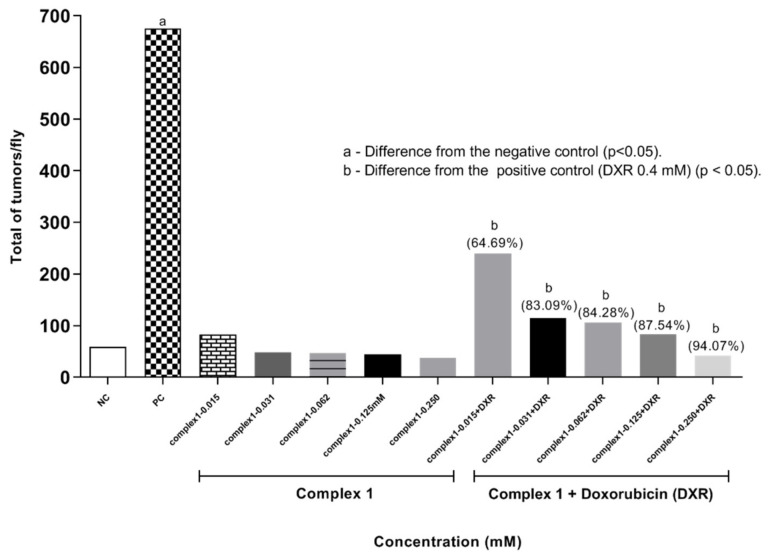
Tumor clones observed in *D. melanogaster*, heterozygote for the *wts* tumor suppressor gene. Flies were treated with complex **1** isolated and associated with Doxorubicin. Statistical diagnosis was performed according to Mann–Whitney Test. Level of significance *p* < 0.05. Percentage (%) represents tumor reduction when complex **1** was associated with DXR. NC: negative control (flies treated with reverse osmosis water). PC: positive control (flies treated with 0.4 mM DXR).

Results for ETT are presented in Figure 4, showing the data for the treatments with complex **1** alone or in combination with DXR 0.4 mM. Frequencies of tumors in the different body segments of *D. melanogaster* treated with positive control (DXR 0.4 mM), negative control (reverse osmosis water) and with different concentrations of complex **1** (0.015, 0.031, 0.062, 0.125, and 0.250 mM) isolated and associated with DXR (0.4 mM) are demonstrated. Larvae exposed only to complex **1** at concentrations of 0.015, 0.031, 0.062, 0.125 and 0.250 mM presented frequencies of 0.41, 0.24, 0.23, 0.22 and 0.18 tumors per fly, respectively. None of the tumor frequencies observed in the treatment with complex **1** showed statistically significant differences from the negative control, according to the Mann–Whitney Test (*p* < 0.05). This result shows the absence of a carcinogenic effect of complex **1** at the concentrations tested. Subsequent malignant neoplasms (SMNs) are known to be one of the most serious and potentially lethal complications of cancer and its therapy. Chemotherapy with platinum analogues has a significant association with the risk of SMN [26], because when they bind to DNA they cause the molecule to twist, inhibiting transcription and causing the death of tumor cells. We suggest that complex **1** does not promote this activity.

The value observed in the negative control was significantly different from that observed in the positive control (DXR 0.4 mM), which was 3.37 tumors per fly. This shows that the lineage responds to tumor induction. The results obtained are compatible with previous studies [21,22,23]. When evaluating the anticarcinogenic action in the co-treatment (simultaneous exposure of complex **1** associated with 0.4 mM DXR), a significant difference was observed for the five concentrations tested (0.015, 0.031, 0.0625, 0.125 and 0.250 mM) compared to the positive control (0.4 mM DXR), according to Mann–Whitney test (*p* < 0.05). The treatments with complex **1** only showed total frequencies of tumors per fly of 1.19, 0.57, 0.53, 0.42 and 0.20, respectively. It is clearly observed that as the concentration of the copper complex increases there was a reduction in the frequency of tumors, reaching levels comparable to the negative control at the highest concentrations of complex **1**. These data show the modulating effect of the complex **1** with the reduction in tumors of 64.69, 83.09, 84.28, 87.54 and 94.07%, respectively. In this context, it is suggested that complex **1** modulates the action of DXR leading to increased damage to cells mutated by DXR, and consequently, to cell apoptosis.

**Figure 5:**
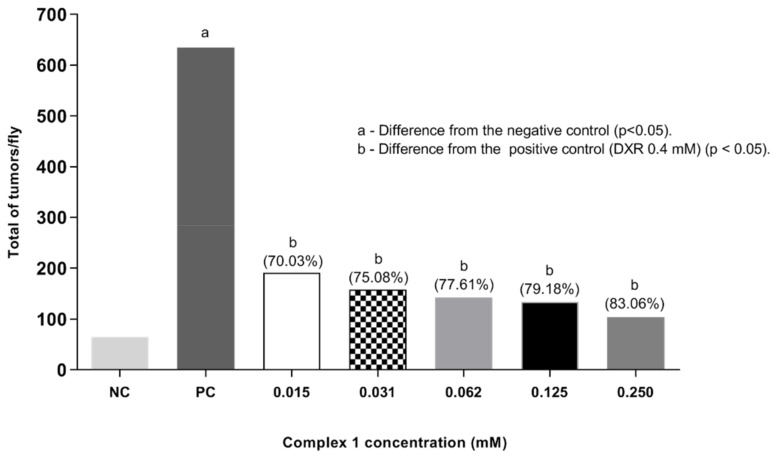
Tumor clones observed in the post-treatment in *D. melanogaster*, heterozygote for the *wts* tumor suppressor gene. Flies were exposed to Doxorubicin (DXR) to induce tumors and, after six hours, to different concentrations of complex **1**. Statistical diagnosis was performed according to the Mann–Whitney Test. Level of significance *p* < 0.05. Percentage (%) represents tumor reduction when complex **1** was associated with DXR. NC: negative control (flies treated with reverse osmosis water). PC: positive control (flies treated with 0.4 mM DXR).

Finally, a post-treatment assay in *D. melanogaster* was also performed under the same experimental conditions as the co-treatment, though exposing the larvae to DXR first. After six hours, the flies were subjected to complex **1**. The results are shown in Figure 5. A significant difference in tumor frequencies was observed at all concentrations tested (0.015, 0.031, 0.062, 0.125 and 0.250 mM) when compared to the positive control (DXR 0.4 mM) according to Mann–Whitney test (*p* < 0.05). The total frequencies of tumors obtained per fly were: 0.95, 0.79, 0.71, 0.66 and 0.52%, respectively. It is noted that, with the increase in the concentration of the copper complex, there was a reduction in the frequency of tumors, which shows the anticarcinogenic potential of the complex and its effect in reducing damage (reduction in tumors of 70.03, 75.08, 77.61, 79.18 and 83.60%, respectively). In this context, the results obtained suggest an antitumor response of complex **1**, which needs to be validated in other animal models.

## 3. Discussion

Herein, the cellular effects of complex **1**, a ternary copper(II) complex containing 4-fluorophenoxyacetic acid hydrazide and 1,10-phenanthroline as ligands was evaluated on prostate cells, and the mutagenic/recombinogenic and anticarcinogenic potential of this compound were recorded in *D. melanogaster*. In the literature, several studies with copper(II) complexes with *N*,*N*-donor ligands (Table 2), such as 1,10-phenanthroline and 2,2-bipyridine, have shown promising antitumor effects [10,27,28,29,30,31,32] and, in our study, complex **1** was selective for the LNCaP lineage, downregulating Ki-67 and Cyclin D1. In PCa, Ki-67 expression has been related to the Gleason score, lower disease-free survival, tumor invasion into the seminal vesicles, and biochemical recurrence or even death after radical prostatectomy [33]. Regarding Cyclin D1, its aberrant expression or amplification promotes the proliferation of malignant prostate cells, which makes it a prognostic marker and a promising therapeutic target [34]. In addition, preliminary studies suggest that the cyclin pathway in PCa plays an important role in the evolution of the disease to a castration-resistant stage, interacting with androgens [35,36]. Therefore, we suggest that complex **1** acts on hormone-responsive cells to prevent their progression to a more advanced stage of the disease, including the castration-resistant phenotype.

**Table 2:** Previous works with copper(II) complexes with *N*,*N*-donor ligands tested in different tumor cells compared with the results obtained.

Complexes	Cell Lines	IC50 (24 h)	Reference
[Cu(dox)(phen)(H_2_O)(ClO_4_)](ClO_4_)	K562	1.93 µM	[27]
[Cu(tc)(phen)(H_2_O)(ClO_4_)](ClO_4_)	K562	2.59 µM	[27]
[Cu(OH-PIP)(Phe)Cl]	CAL-51	0.52 µM	[28]
[Cu(OH-PIP)(Phe)Cl]	MDA-MB-231	18.89 µM	[28]
[Cu(OH-PIP)(Phe)Cl]	MCF-7	30.88 µM	[28]
[Cu(dox)(phen)]^2+^	B16F10	1.4 µM	[29]
[Cu(dox)(phen)]^2+^	Sarcoma TG180	6.2 μM	[29]
[Cu(dox)(phen)]^2+^	Sarcoma S180	13.3 μM	[29]
bis[(µ2-chloro)chloro(1,10-phenanthroline)copper(II)]	MDA-MB-32	0.65 μM	[30]
bis[(µ2-chloro)chloro(1,10-phenanthroline)copper(II)]	HT-29	0.60 μM	[30]
bis[(µ2-chloro)chloro(1,10-phenanthroline)copper(II)]	A549	0.85 μM	[30]
bis[(µ2-chloro)chloro(1,10-phenanthroline)copper(II)]	B16F10	0.91 μM	[30]
[Cu(bta)(1,10-phenanthroline)(ClO_4_)]	Sarcoma TG180	7.4 μM	[31]
[Cu(tdp)(phen)]^+^	MCF-7	1.6 μM	[32]
[Cu(tdp)(phen)]^+^	MDA-MB-231	1.9 μM	[32]
[Cu(4-FH)(phen)(ClO_4_)_2_]	K562	1.8 μM	[10]
[Cu(4-FH)(phen)(ClO_4_)_2_]	K562	26.2 μM	[10]
[Cu(4-FH)(phen)(ClO_4_)_2_]	K562	1.6 μM	[10]
[Cu(4-FH)(phen)(ClO_4_)_2_]	K562	28 μM	[10]
[Cu(4-FH)(phen)(ClO_4_)_2_]	K562	15 μM	[10]
Complex **1**	PC3	45.58 μM	Present paper
Complex **1**	LNCap	9.05 μM	Present paper

In toxicity studies, in vivo tests may simulate what happens systematically. Indeed, to avoid overuse of mammals, *D. melanogaster* is a validated model for toxicological assays, since about 80% of the genes associated with human diseases have homologues in these flies [37,38]. Our results suggest a possible selectivity of complex **1**, since no mutagenic/recombinogenic effects were observed in somatic cells of *D. melanogaster* using the SMART test. González et al. [39] emphasized that some copper complexes may effectively kill cancer cells without showing mutagenic activity, which decreases the incidence of SMNs. In fact, the ideal drugs for the cancer treatment should not cause damage to normal cells, but they must, at the same time, make tumor cells unviable [40]. Complex **1** meets these criteria, which makes it a possible anticancer agent.

Although DXR is a potent anticancer compound, it is responsible for related side effects [41]. In our flies treated with DXR (positive control) a high frequency of mutagenic/recombinogenic events and epithelial tumors was observed. Therefore, the present results are pioneering in demonstrating the antitumoral profile of complex **1** in *D. melanogaster*, which is an organism capable to activate, enzymatically, promutagens and procarcinogens in vivo [42].

According to Ahmad et al. (2018), copper(II) complexes have potentially effective anticancer activity in vivo, and drugs should be evaluated for their ability to reduce tumors [43]. For this, we performed the ETT test in the co-treatment (complex **1** + DXR) and post-treatment assays and we demonstrated the anticarcinogenic and DXR modulator potential of complex **1**. It is well known that DXR-induced toxicity challenges the chemotherapy. We suggest that complex **1** may be an adjuvant or used in combination with DXR, decreasing its toxicity without impairing efficacy. Additionally, other markers must be evaluated, including Topoisomerases. It is known that the main mechanism of action of DXR is by inhibiting topoisomerases [41]. These are vital enzymes in cell proliferation, and targeting them causes DNA damage, and ultimately cell death. Previous studies demonstrated the action of some copper(II) complexes through the inhibition of Topoisomerase I causing DNA double-strand break [44,45,46]. Thus, it can be inferred that complex **1** may have potentiated the damage induced by DXR in *D. melanogaster* cells, activating apoptosis and preventing the expression of the mutant phenotype.

## 4. Materials and Methods

### 4.1. Cu(II) Complex and Starting Materials

The complex **1** was prepared according to the published procedure [10]. Herein, elemental analysis data (CHN) were used to verify the purity of **1**. All chemicals were purchased from Merck (Kenilworth, NJ, USA) and were used as received unless otherwise stated. DXR was used in the present study as a positive control at 0.4 mM (diluted in autoclaved reverse osmosis water) for in vivo tests. DXR at 0.4 mM was previously able to generate ROS and induce homologous recombination in *Drosophila melanogaster* through topoisomerase inhibition [21,47,48,49].

### 4.2. Cell Lines

Three prostate cell lines PNT-2 (non-tumorigenic), LNCaP (hormone-responsive PCa) and PC-3 (androgen-independent PCa) were maintained in RPMI-1640 (Sigma-Aldrich, St. Louis, MO, USA), supplemented with 10% fetal bovine serum (FBS) (Gibco; Thermo Fisher Scientific, Waltham, MA, USA) and 50 µg/mL gentamicin (Cultilab, Campinas, Brazil). The lineages were incubated at 37 °C with 5% CO_2_ and the culture medium was changed on alternate days. Upon reaching 80% confluence, cells were seeded for subsequent assays. Cells were purchased from the American Type Culture Collection (ATCC, Manassas, VA, USA), authenticated and routinely checked for mycoplasma contamination.

### 4.3. MTT Assay

Cytotoxicity of complex **1** was assessed by MTT assay (Sigma-Aldrich) [50] with some modifications. PNT-2 (2.0 × 10^4^), LNCaP (1.5 × 10^4^), and PC-3 (1.5 × 10^4^) cells were seeded in 96-well plates and, after confluence, were treated with different concentrations of complex **1** (1 µM, 5 µM, 10 µM, 12.5 µM, 25 µM, and 50 µM), diluted in 0.5% dimethyl sulfoxide (DMSO). The experiment was conducted at 24 and 48 h. Wells with untreated cells were used as a viability control, and cells treated with DMSO alone were included as diluent control. The metabolically active cells reduced the MTT (5 mg/mL) to formazan crystals, which were dissolved in DMSO 0,5%. Absorbance measurements of each well were taken at 570 nm (Thermo Plate TP-Reader, Thermo Fisher Scientific, Waltham, Massachusetts, USA). The cell viability was expressed as a percentage of the control and IC50 was calculated by non-liner regression. The SI was calculated from the ratio between the IC50 values of the non-tumorigenic cell line per the tumorigenic cell line and considered significant when SI ≥ 2 [51].

### 4.4. Flow Cytometry

In order to analyze the expression of Ki-67 and Cyclin D1, LNCaP cells were treated for 48 h with complex **1** at a concentration of 6.5 µM (determined from the IC50 value). After treatment, cells were washed with PBS (Phosphate-Buffered Saline), and permeabilized by BD Cytofix/Cytoperm™ Fixation/Permeabilization Solution Kit (BD Pharmingen, San Jose, CA, USA). Subsequently, LNCaP cells were stained with anti-Ki67 (1:100; RM360, Sigma) and anti-Cyclin D1 (1:100; ab10540, Abcam, Cambridge, UK) for 1 h at room temperature. Anti-rabbit IgG-FITC (1:200, 656111, Thermo Fisher Scientific) and anti-mouse IgG-Atto 647N (1:50, 50185, Sigma Aldrich) were used as a secondary antibody, respectively. Cell staining was analyzed using flow cytometry (Accuri C6, BD Pharmingen).

### 4.5. Statistical Analysis

The GraphPad Prism 8.0 software (GraphPad Software Inc., La Jolla, California, USA) was used to calculate the statistical significance of the assays. Data normality was verified using Kolmogorov–Smirnov’s test. Differences between cells subjected to the same treatment in the MTT assay were calculated by the One-Way ANOVA followed by Tukey’s test. Student’s *t*-test was used for the flow cytometry experiments. Three independent assays were performed in triplicate and *p* < 0.05 was considered as significant.

### 4.6. Toxicity Test in D. melanogaster

For in vivo assays, serial dilutions (0.015, 0.031, 0.062, 0.125 and 0.250 mM) of complex **1** were prepared using reverse osmosis water. *D. melanogaster* specimens collected in this experiment were preserved and handled at LABCIM (Laboratory of Cytogenetic and Mutagenesis of the University Center of Patos de Minas—UNIPAM). The lineages of *D. melanogaster* were kept inside a B.O.D. incubator at 25 °C and 60% of humidity with a photoperiod of 12 h. The toxicity test was performed to define the concentrations to be used in SMART and ETT tests. Thirty heterozygous larvae *wts*+/+*mwh*, from the cross between virgin females of *wts*/*TM3*, Sb^1^ and males *mwh*/*mwh*, were grown in medium containing the compound complex **1** at concentrations of 0.015, 0.031, 0.062, 0.125, 0.250, 1.00, 2.00, 4.00 and 8.00 mM.

According to Spanó et al. (2001) the larvae were exposed to the culture medium containing the complex **1** and mashed potatoes (Yoki^®^ Alimentos S.A., Sao Paulo, Brazil), feeding for 48 h (chronic treatment). At the end of the entire development phase, which lasts approximately one week, the emerging flies were preserved in 70% alcohol. Subsequently, the individuals were counted in a stereoscopic microscope. A survival curve was then constructed in order to establish the toxicity of the compound based on the percentage of flies surviving the treatment. It was also possible to determine the lethal dose of complex **1** for *D. melanogaster*. Statistical significance (*p* < 0.05) was determined using the Chi-square test, with the GraphPad Prism 6.0 software (GraphPad Software Inc.) [17].

### 4.7. SMART Test in D. melanogaster Wings

As established by Graf and van Schaik [52] for the SMART test, the mutant lineages of *D. melanogaster* used were kindly supplied by Dr. Urich Graf, of the Toxicology Institute, University of Zurich, Shwerzenbach, Switzerland. The test uses three lineages, *mwh*, *flr^3^* and *ORR*, which possess the genetic markers multiple wing hairs (*mwh*, 3-0.3) and *flare-3* (*flr^3^*, 3-38.8). Two crosses were performed: the ST Cross, in which virgin females *flr^3^/In(3LR)TM*, *ri p^p^ sepI(3)89Aa bx^34e^* and *Bd^s^* are crossed with males *mwh/mwh*; and the HB Cross, in which virgin females *ORR*; *flr^3^/In(3LR)TM, ri p^p^ sepI(3)89Aa bx^34e^* and *Bd^s^* are crossed with males *mwh/mwh*.

The crosses mentioned above produced two types of progeny: (i) trans-heterozygous individuals (MH) for the marker genes; and (ii) balancer-heterozygous individuals (BH) [53,54]. BH individuals are phenotypically differentiated from MH individuals by the presence of indentations on the edge of the wings, a characteristic conferred by the *TM3* marker that leaves them with a serrated aspect [16].

The experiment was conducted according to the protocol described by Graf et al. [16]. The collection of *ORR* and *flr^3^* virgin females was performed at 2-h intervals, between 9 a.m. and 5 p.m. After this stage, 100 females of each lineage (*ORR* and *flr^3^*) were placed together with 50 males (*mwh*) for the mentioned crossings. The eggs were laid for 8 h in flasks containing a solid base of agar (4% agar in water) and a layer of yeast (*Sacharomyces cerevisae*) supplemented with sugar. After 72 h of oviposition, third-stage larvae were washed with reverse osmosis water and collected with the aid of a fine mesh steel sieve. Inside the fume hood, serial dilutions of the complex **1** and DXR were performed.

Thereafter, chronic treatment was conducted in which the aforementioned larvae were transferred to 25 mL flasks containing 5 mL of alternative medium (mashed potatoes), associated with complex 1 in the concentrations of 0.015, 0.031, 0.062, 0.125 and 0.250 mM. Reverse osmosis water was used as a negative control and DXR (0.4 mM) as a positive control.

After this procedure, the flies fed on the medium and, a week later, completed the stages of development (metamorphosis). Then the adult individuals were collected and kept in 70% ethanol. Subsequently, the wings of the collected flies were detached using entomological forceps under a stereoscopic microscope and placed in pairs on histological slides, with 5 pairs of female wings at the top of the slide and 5 pairs of male wings at the bottom. The wings were fixed with Faure solution (50 mg of gum arabic, 30 g of chloral hydrate, 30 mL of glycerol, 50 mL of ultrapure water). The analysis of the wings was performed under a light microscope, at a magnification of 400x, recording the number, types, size and position of the spots. The trichomes present on the dorsal and ventral surface of the wings were observed in order to identify mutant hair spots classified as simple (*mwh* or *flr^3^*) or twin (*mwh* and *flr^3^*), and as small (1 to 2 mutant cells) or large (with more than 3 mutant cells). Sections (A, B, C, C’, D, D’ and E) were used to record the scale of each spot.

The statistical analysis of the experiment was carried out using the conditional binomial test of [55], at a significance level of 5%. The procedure proposed by Frei and Würgler [25] was used for the analysis of multiple decisions, generating four different diagnoses: positive, weak positive, negative or inconclusive.

Based on the frequency of induction of clones per 10^5^ cells, the recombinogenic activity was calculated as: Frequency of mutation (FM) = frequency of clones in BH flies/frequency of clones in MH flies. Frequency of recombination (FR) = 1 − FM [56].

### 4.8. ETT Test in D. melanogaster

ETT has been used to evaluate the carcinogenic or anticarcinogenic activity of different compounds/substances. This test allows the evaluation of simple and combined assays, in co-treatment and post-treatment strategies. In co-treatment, the larvae are simultaneously exposed to DXR and the compound tested, whereas in the post-treatment the larvae are previously induced to the tumor and, shortly after 6 h, they are exposed to the substance under study, in order to verify the reversal of damages [42].

Two mutant lineages of *D. melanogaster* were used, including virgin females *wts/TM3*, *Sb^1^* and males *mwh/mwh*. The collection of virgin females (*wts/TM3*, *Sb^1^*) and males (*mwh/mwh*) was carried out for three consecutive days, in flasks containing standard culture medium. On the last day, the two lineages were placed together for crossing. About 48 h after this period, males and females were placed in flasks containing a culture medium appropriate for laying (yeast and sugar), where the females laid their eggs. The 72 h larvae were fed with culture medium containing the complex **1** at the concentrations of 0.015; 0.031; 0.062; 0.125 and 0.250 mM (in quadruplicate), chosen based on the toxicity test result. The assay was performed with complex **1** alone or combined with DXR (0.4 mM). The entire procedure was carried out under aseptic and controlled conditions. The reverse osmosis water was used as a negative control and DXR (0.4 mM) as a positive control. Adult flies were collected and kept in ethanol (C_2_H_6_O) 70%.

The entire body of adult flies was analyzed under a stereoscopic microscope with the aid of entomological forceps, excavated plaque and glycerin. The identification and selection of the individuals used in the analyses were based on the characteristic of the body and head hairs of the flies. Adult flies that had the chromosomal balancer (*TM3*, *Sb^1^*) has the *wts*+/+*TM3* genotype, which have a short and thick hair phenotype, were discarded. Individuals with long and thin hairs and genotype (*wts*+/+*mwh*) were analyzed due to the presence of the gene under study (*wts*), homologous to large tumor suppressor kinase 1 (*LATS1*) tumor suppressor in humans [57]. The statistical analysis of the ETT test was performed using the Mann–Whitney test, with the Prophet software version 2019 Q2, at a significance level of 5%.

## 5. Conclusions

Complex **1** was selective to hormone-responsive PCa cells and did not induce mutagenicity/recombinogenicity and carcinogenicity in our in vivo model. Additionally, complex **1** presented anticarcinogenic potential and is a DXR modulator, with desirable characteristics for a chemotherapeutic agent. Therefore, we suggest complex **1** as a promising antitumoral agent. This compound should be widely studied in other tumor types and experimental models to better elucidate its mechanism of action, antitumoral effect, safety and selectivity.

## Figures and Tables

**Table 1:** Results obtained in the marked trans-heterozygous (MH) descendants of *D. melanogaster* derived from the standard (ST) cross and high-bioactivation (HB) cross treated with different complex **1** concentrations, negative control (reverse osmosis water) and positive control (0.4 mM Doxorubicin).

Treatments		Number of Flies (*n)*	Spots per Fly (N^°^ of Spots); Statistical Diagnosis ^a^												Spots with *mwh* Clone ^c^ (*n*)	Frequency of Clone Formation/ 10^5^ Cells per Cell Division ^d^	
DXR (mM)	Complex 1 (mM)		Small Single (1–2 Cells) ^b^ *m* = 2			Large Single (>2 Cells) ^b^ *m* = 5			Twin *m* = 5			Total Spots *m* = 2				Observed	Control Corrected
*mwh/flr^3^*																	
*ST Cross*																	
0	0	60	0.40	(24)		0.03	(2)		0.02	(1)		0.45	(27)		27	0.92	
0.4	0	60	2.62	(157)	+	4.52	(271)	+	5.33	(320)	+	12.47	(748)	+	697	23.80	22.80
0	0.015	60	0.32	(19)	-	0.03	(2)	i	0.08	(5)	i	0.43	(26)	-	23	0.79	−0.14
0	0.031	60	0.35	(21)	-	0.05	(3)	i	0.02	(1)	i	0.42	(25)	-	23	0.79	−0.14
0	0.062	60	0.35	(21)	-	0.03	(2)	i	0.03	(2)	i	0.42	(25)	-	25	0.85	−0.07
0	0.125	60	0.23	(14)	-	0.05	(3)	i	0.12	(7)	i	0.40	(24)	-	21	0.72	−0.20
0	0.250	60	0.25	(15)	-	0.00	(0)	i	0.12	(7)	i	0.37	(22)	-	16	0.55	−0.38
*HB Cross*																	
0	0	60	0.62	(37)		0.20	(12)		0.00	(0)		0.82	(49)		48	1.64	
0.4	0	60	1.30	(78)	+	1.10	(606)		1.98	(119)	+	13.38	(803)	+	782	26.71	25.07
0	0.015	60	0.27	(16)	-	0.13	(8)	-	0.03	(2)	i	0.43	(26)	-	26	0.89	−0.75
0	0.031	60	0.30	(18)	-	0.12	(7)	-	0.02	(1)	i	0.43	(26)	-	26	0.89	−0.75
0	0.062	60	0.38	(23)	-	0.03	(2)	i	0.00	(0)	i	0.42	(25)	-	25	0.85	−0.89
0	0.125	60	0.22	(13)	-	0.10	(6)	-	0.07	(4)	i	0.38	(23)	-	23	0.79	−0.85
0	0.250	60	0.10	(6)	-	0.10	(6)	-	0.00	(0)	i	0.20	(12)	-	12	0.41	−1.23

Marker-trans-heterozygous flies (*mwh*/*flr^3^*) were evaluated. ^a^ Statistical diagnosis according to Frei and Würgler [25]: +, positive; -, negative; i, inconclusive. m = multiplication factor for significantly negative results. Level of significance *p* < 0.05. ^b^ Including rare single *flr^3^* spots. ^c^ Considering the *mwh* clones for the single spots and *mwh* for the twin spots. ^d^ Frequency of clone formation: clones/flies/48,800 cells (without size correction).

## Data Availability

Not applicable.

## Abbreviations

**BH**	**Balancer-heterozygous individuals**
**DXR**	**Doxorubicin**
**LD**	**Lethal Dose**
**ETT**	**Epithelial tumor test**
**FM**	**Frequency of mutation**
**FR**	**Frequency of recombination**
**HB**	**High Bioactivation cross**
***LATS1***	**Large tumor suppressor kinase 1**
**MH**	**Trans-heterozygous individuals**
**MTT**	**3-(4,5-dimethylthiazol2-yl)-2,5-diphenyltetrazolium bromide**
**PCa**	**Prostate Cancer**
**ROS**	**Reactive oxygen species**
**SI**	**Selectivity Index**
**SMART**	**Somatic Mutation and Recombination Test**
**SMN**	**Subsequent malignant neoplasm**
**ST**	**Standard cross**
**TX**	**Toxicity Test**
